# RadDeploy: A framework for integrating in-house developed software and artificial intelligence models seamlessly into radiotherapy workflows

**DOI:** 10.1016/j.phro.2024.100607

**Published:** 2024-07-02

**Authors:** Mathis Ersted Rasmussen, Casper Dueholm Vestergaard, Jesper Folsted Kallehauge, Jintao Ren, Maiken Haislund Guldberg, Ole Nørrevang, Ulrik Vindelev Elstrøm, Stine Sofia Korreman

**Affiliations:** a Danish Centre for Particle Therapy, Aarhus University Hospital, Palle Juul-Jensens Boulevard 25, 8200 Aarhus N, Denmark; b Department of Oncology, Aarhus University Hospital, Palle Juul-Jensens Boulevard 35, 8200 Aarhus N, Denmark; c Department of Clinical Medicine, Aarhus University Hospital, Palle Juul-Jensens Boulevard 99, 8200 Aarhus N, Denmark

**Keywords:** Radiotherapy, In-house, Deploy, Container, DAG, Artificial intelligence

## Abstract

The use of and research in automation and artificial intelligence (AI) in radiotherapy is moving with incredible pace. Many innovations do, however, not make it into the clinic. One technical reason for this may be the lack of a platform to deploy such software into clinical practice. We suggest RadDeploy as a framework for integrating containerized software in clinical workflows outside of treatment planning systems. RadDeploy supports multiple DICOM as input for model containers and can run model containers asynchronously across GPUs and computers. This technical note summarizes the inner workings of RadDeploy and demonstrates three use-cases with varying complexity.

## Highlights

• RadDeploy is a deployment platform for in-house containerized software and AI-models.
• RadDeploy offers a comprehensive DICOM-based triggering of flows.
• Flows can have multiple DICOM series as input.
• Flows can be designed as directed acyclic graphs of docker containers.
• RadDeploy scales across multiple GPUs and computers.

## 1 Introduction

The clinical use of and research in computers for automation and artificial intelligence (AI) in radiotherapy is moving with incredible pace. AI is currently revolutionizing auto-segmentation of organs-at-risk [1], [2], [3], [4], [5] and holds great promise for auto-segmentation of target structures [6], [7], [8], [9], dose prediction [10], [11], [12], [13], [14], outcome prediction [15] and many other complex tasks in radiotherapy. While some of these technologies are available from commercial vendors, the cutting edge of research is not. Furthermore, commercial products might not comply with local standards and preferences. These issues are sometimes solved with in-house developed tools.

Many innovations in healthcare do not make it into clinical practice [16]. In radiotherapy, a technical reason for this may be the lack of a platform to deploy the in-house developed software. Many treatment planning systems (TPSs) have scripting capabilities, through which some software can run. Using such tools might lead to implementations being tightly coupled to the ecosystem of that particular TPS and other local IT infrastructures. Hence, if a piece of software is successfully implemented locally it might not be transferable to other institutions which may inhibit development and validation of the tool.

The challenge of executing software reproducibly in different computational environments is not confined to radiotherapy. During the last decade, *containers* have revolutionized the way software is developed and deployed [17]. Containers can be thought of as “frozen” computer environments which execute the same way on different computers [18]. While containers solve the reproducibility issue, they do, however, not offer a solution for implementation − this must be tailored to the specific domain.

We propose RadDeploy as a simple-to-configure, yet flexible and scalable framework to facilitate seamless implementations of in-house developed software encapsulated in containers. The aim of this work was to investigate the usability of RadDeploy for deploying three in-house developed AI-models with varying complexity in clinical practice.

## 2 Material and methods

### 2.1 Terminology

In RadDeploy, a *model* refers to a unit of software which is encapsulated in a Docker container [19]. A model could be an in-house developed AI model for auto-segmentation, dose prediction or optimization but, in fact, any piece of software that can be wrapped in a Docker container qualifies as a model.

A *flow* refers to the passage of a set of DICOM files through the system. A flow consists of three major parts: *triggers*, *models* and *destinations*. *Triggers* define DICOM tag values which, when found in the received files, will trigger the flow. *Models* define which and how Docker containers should be executed. *Destinations* specify DICOM nodes to which outputs should be sent.

### 2.2 Architecture and services

RadDeploy consists of microservices each dedicated to do isolated tasks. Services communicate through message queues provided by a message broker. Hence, services receive messages from specified queues, perform some operations, and publish results to other queues. More details may be found in the repository wiki [20].

#### 2.2.1 Storage service class provider (SCP)

**Fig. 1:**
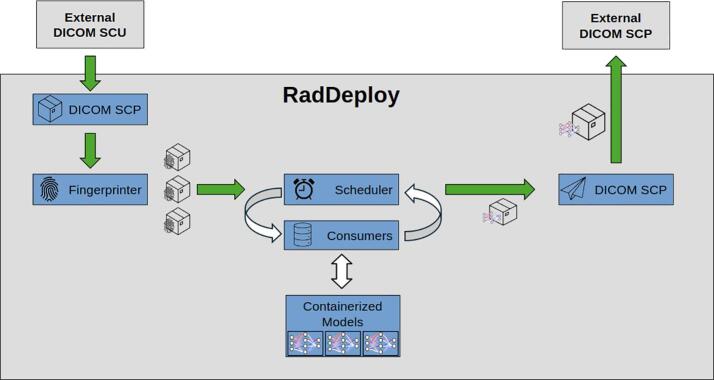
Schematic representation of RadDeploy. Gray boxes represent logical computer units and blue boxes represent services. Arrows indicate the message flow through the stack. Note that under the hood, messages published to and subscribed from the underlying message broker (not shown) and files are posted and retrieved from a file storage-service (not shown). (For interpretation of the references to colour in this figure legend, the reader is referred to the web version of this article.)

The SCP is the entrypoint for DICOM files into RadDeploy (Fig. 1). It acts like any other SCP to which DICOM files can be sent from TPSs and alike. It puts all received files in a tarball [21] (type of archive file) and sends it to a file storage service (not to be discussed further). Subsequently, the SCP publishes a unique identifier (UID) of the tarball.

#### 2.2.2 Fingerprinter

This service subscribes to messages coming from the SCP. When it receives a message, it determines which of the DICOM files from the tarball are eligible for which flows. When a set of files are found to match the triggers for a particular flow, a new tarball containing only the relevant files is sent to the file storage and the corresponding tarball UID is published along with the flow definition (see section 2.1).

#### 2.2.3 Scheduler

This service receives messages from the fingerprinter and consumers (see section 2.2.4). It orchestrates the queuing of models eligible for execution and determines when a flow is finished. It publishes finished flows and the UID of the resulting tarball.

#### 2.2.4 Consumer

This service handles an arbitrary number of workers, whose purpose is to execute models as specified in the flow definition. Messages are retrieved from CPU/GPU queues to which the scheduler publishes.

#### 2.2.5 Storage service class user (SCU)

The SCU subscribes to finished flows published by the scheduler. It sends all DICOM files contained in the resulting tarball to the DICOM nodes (SCPs) defined in *destinations* in the flow definition. Such destinations are usually TPSs, Picture Archiving and Communication Systems (PACSs) or even the SCP of RadDeploy itself.

### 2.3 Deployment

RadDeploy can be deployed with a Docker compose file (supplementary figure S1). For this, the host machine must have the Docker engine installed with linux as the backend. If models require GPU acceleration, NVIDIA drivers and the nvidia-container-toolkit [22] must be installed. An example compose file can be downloaded off Gitlab and RadDeploy may be spun up with a single command in a terminal. A detailed guide can be found in the Wiki of the RadDeploy repository [20].

## 3 Results

At the time of writing, RadDeploy is a relatively young platform. However, it has already proven capable in a variety of tasks. In this section, we present three use-cases of increasing complexity and demonstrate how RadDeploy integrates these in our clinical/research environment.

### 3.1 Single modality auto-segmentation

Auto-segmentation models are often executed on a single image modality such as CT or MRI. In our clinic, we run several in-house developed auto-segmentation models to produce structures that are used as templates for clinical organs-at-risk contouring. When a patient is scanned, the DICOM image files are automatically exported from the CT/PET or MRI scanners to a standalone server running RadDeploy. Here, relevant flows are triggered and the resulting DICOM Radiotherapy Structure Sets (RTSS) are sent to the TPS. An example of the flow definition for this may be found in supplementary figure S2.

As a further example, the wiki provides a guide on how to setup RadDeploy to run TotalSegmentator [23] for single modality auto-segmentation [24].

### 3.2 Multi modality auto-segmentation

In multi modality auto-segmentation several co-registered images are used to predict contours. Multi modality auto-segmentation is generally not offered commercially and are therefore often in-house developments.

A prospective randomized controlled trial is being conducted at our institution to evaluate the usefulness of deep learning contours as templates for delineation of primary and nodal gross tumor volumes (GTV-T and −N) of head-and-neck cancer patients [25]. The model input is four imaging modalities (CT, PET, T1- and T2-weighted MRI) which are manually pushed to RadDeploy from Aria Eclipse (Varian Medical Systems, Palo Alto, CA, USA). The model consists of multiple steps. Firstly, MRIs are deformably registered to the CT, secondly, the GTVs are predicted, and thirdly, a randomization determines if the GTV predictions or empty structures are sent to the TPS for clinical contouring. Additionally, patient identifiers and model data is pushed to a RedCap database via the HTTP API. An example of the flow definition may be found in supplementary figure S3.

### 3.3 Synthetic CT generation

Proton radiotherapy is highly sensitive to anatomical changes which may arise during the period of treatment [26], [27]. Daily cone beam CTs (CBCTs) are obtained at the gantry for the purpose of patient positioning and evaluation of anatomical changes. Unfortunately, the image quality of CBCTs is often insufficient for performing proton dose calculations accurate enough to assess the need for replanning. Therefore, the quality of proton dose calculations on synthetic CT scans (sCT) made from daily CBCT scans is currently being investigated at our department using an in-house developed deep learning model.

RadDeploy triggers the flow when receiving a planning CT, a CBCT and a rigid registration file. The output is a DICOM sCT containing an inferred anatomy of the patient. An example of the flow definition may be found in supplementary figure S4.

## 4 Discussion

In this technical note, we have presented RadDeploy which is a framework for deploying containerized software seamlessly into clinical workflows. We have demonstrated three use-cases which vary in complexity and maturity and RadDeploy has proven able to perform stably across these.

Apart from RadDepoy, at least one other framework exists for integrating containerized models [28]. While the purpose is the same, it is achieved differently. Hence, RadDeploy should be seen as an alternative solution with its own set of pros and cons.

A unique feature of RadDeploy is the microservice architecture. Microservices provide a high degree of decoupling. As long as the structure of messages that are flowing between the services is strictly followed, maintenance of existing and development of new services are possible without interfering with the rest of the system. Furthermore, services like the SCP, fingerprinter, consumer and SCU are stateless, and may therefore be scaled horizontally across multiple computers and GPUs. RadDeploy can easily be extended with new services providing additional functionality as these can seamlessly be “mounted” into the stack by subscribing/publishing to specific queues. A notable disadvantage of the microservice architecture is that there is no central database or service that knows the entire state of the system. This increases the risk of messages being lost unnoticed due to power outage or network failure. Furthermore, it is essentially impossible to test every state of the system as messages flow asynchronously between services.

Although not demonstrated here, flows may be designed as Directed Acyclic Graphs (DAGs) represented as models being nodes and in- and output mounts being edges. When flows are designed as DAGs, model containers are scheduled for execution asynchronously when the required inputs are available. This would be useful in the multi-modality auto-segmentation flow as it contains two time consuming and computationally heavy tasks: (1) CPU-based image registration, and (2) GPU-based five-fold prediction of target structures. Hence, the current all-in-one container could be decomposed into a CPU container and five GPU containers, which would be executed asynchronously and potentially across multiple computers/GPUs and thereby ensuring better utilization of computational resources. It is, however, not done currently for the trial, as the model container was developed before DAG scheduling was implemented in RadDeploy. An illustrative flow definition of DAG scheduling can be found in supplementary figure S5.

**Fig. 2:**
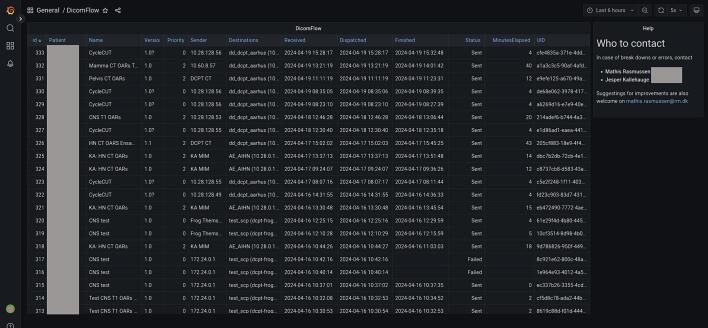
An example of a Grafana dashboard made from the database of the scheduler service.

There is still room for improvement of RadDeploy, and future developments might include a graphical user interface for setup, configuration and maintenance. Currently, the database of the scheduler can be visualized using a dashboard service like Grafana [29] (Fig. 2) which is useful for end-users to keep track of flows but it does not provide any features for controlling jobs. Furthermore, distributed DAG scheduling is a complex task which in the future might be handled better by a third party tool such as Argo Workflows [30], Apache AirFlow [31] or Celery [32].

All stable versions of RadDeploy are made available as public Docker images in the Gitlab container registry [20]. Thus, it is possible to set up the default stack within minutes. Thereby, resources can be put into development, testing, validation and documentation of models without worrying about deployment; as long as the software can be wrapped in a Docker container and has DICOM files as input, RadDeploy can be used. If the default stack does not accommodate the research/clinical requirements, RadDeploy is open source and may freely be modified and extended with new services. Feature requests, requests for support and contributions to the main repository and the wiki are also warmly welcome.

In conclusion, RadDeploy is a microservice-based framework which allows deployment of in-house developed software as Docker containers in radiotherapy workflows. It offers a comprehensive triggering functionality which enables execution of multi-modality models. Furthermore, flows can contain multiple model containers as directed acyclic graphs which allows asynchronous model execution across multiple GPUs and computers.

## Funding

This work is funded with salary for MER from Aarhus University and Aarhus University Hospital.

## CRediT authorship contribution statement

**Mathis Ersted Rasmussen:** Conceptualization, Methodology, Software, Writing – original draft, Visualization, Project administration. **Casper Dueholm Vestergaard:** Software, Methodology, Validation, Writing – original draft. **Jesper Folsted Kallehauge:** Conceptualization, Methodology, Software, Validation. **Jintao Ren:** Methodology, Writing – original draft, Visualization. **Maiken Haislund Guldberg:** Investigation, Software. **Ole Nørrevang:** Writing – original draft, Funding acquisition. **Ulrik Vindelev Elstrøm:** Methodology, Validation. **Stine Sofia Korreman:** Conceptualization, Writing – original draft, Supervision, Project administration, Funding acquisition.

## Declaration of Competing Interest

The authors declare that they have no known competing financial interests or personal relationships that could have appeared to influence the work reported in this paper.
