# Supplementary material for: RadDeploy: A framework for integrating in-house developed software and artificial intelligence models seamlessly into radiotherapy workflows
Source: Phys Imaging Radiat Oncol. 2024 Jul 2;31:100607. doi: 10.1016/j.phro.2024.100607 (PMC11283118; doi:10.1016/j.phro.2024.100607)
Supplement: Supplementary Data 1 [file mmc1.docx]

# **Supplementary Material**

# A note on the project name

This project was previously named “DicomFlow”, but the authors became aware that it collided with existing projects. The name was therefore changed, but the old name might still occur until the entire project is refactored.

## Supplementary figure S1: Docker Compose file to deploy RadDeploy

A simplified version of the docker-compose.yaml file used to deploy the core of RadDeploy. Along with the five core services described in materials and methods, there is the message broker (rabbit) and the file storage-service. Compose files are developed and documented by Docker (https://docs.docker.com/compose/)

| # Slightly simplified version. A full example may be found at:  # https://gitlab.com/aarhus-radonc-ai/repos/raddeploy/-/blob/dd07568879d8e29d4931b60227c46c4ab1113b4f/examples/RadDeploy/docker-compose.yaml  services:  rabbit:  image: rabbitmq:3-management  volumes:  - ${RABBIT_CONF_DIR}/timeout.conf:/etc/rabbitmq/conf.d/timeout.conf   mysql:  image: mysql:latest   storescp:  image: registry.gitlab.com/aarhus-radonc-ai/repos/raddeploy/  storescp:latest  ports:  - "10000:10000"    fingerprinter:  image: registry.gitlab.com/aarhus-radonc-ai/repos/raddeploy/  fingerprinter:latest  volumes:  - ./flows:/opt/RadDeploy/fingerprinter/flows     scheduler:  image: registry.gitlab.com/aarhus-radonc-ai/repos/raddeploy/  scheduler:latest  environment:  - SCHEDULER_DATABASE_URL=mysql+pymysql://root:password@mysql:3306/mysql    consumer:  image: registry.gitlab.com/aarhus-radonc-ai/repos/raddeploy/  consumer:latest  environment:  - GPUS=0 # Comment out if you don't have a GPU installed  - CPUS=1  volumes:  - /var/run/docker.sock:/var/run/docker.sock   storescu:  image: registry.gitlab.com/aarhus-radonc-ai/repos/raddeploy/  storescu:latest    file-storage:  image: registry.gitlab.com/aarhus-radonc-ai/repos/raddeploy/  file_storage:latest  reverse-proxy:  image: traefik:v2.6  command:  - --providers.docker=true  - --entrypoints.websecure.address=:443  - --providers.file.watch=true  ports:  - 443:443  labels:  traefik.enable: true  volumes:  - /var/run/docker.sock:/var/run/docker.sock   dashboard:  image: grafana/grafana-enterprise  labels:  - "traefik.enable=true"  - "traefik.http.routers.grafana.rule=Host(`localhost`)"  - "traefik.http.services.grafana.loadbalancer.server.port=3000"  - "traefik.http.routers.grafana.tls=true"  volumes:  - config:/etc/grafana:rw  - home:/usr/share/grafana:rw  - data:/var/lib/grafana:rw  volumes:  config:  home:  data: |
| --- |

## Supplementary figure S2: Single modality auto-segmentation

| name: "HN CT OARS Ensemble" # Arbitrary name version: "1.1" # Arbitrary name priority: 2 # Priority of this flow.   # List with a single trigger. DICOM files which match the rules will be # sent to the specified docker container (model) triggers:  - SOPClassUID: ["1.2.840.10008.5.1.4.1.1.2"] # SOPClassUID must contain...  StudyDescription: ["Neck|Cerebrum|CNS"] # StudyDescription must contain   # Neck or Cerebrum or CNS  SeriesDescription: ["~AIHN", "~CycleCUT"] # SeriesDescription must not # contain "AIHN" or "CycleCUT"  Manufacturer: ["Siemens"] # Manufacturer must contain # "Siemens" models:  # keyword arguments to specify behaviour of docker container. This is a   # wrapper of docker-py's run command:   # "https://docker-py.readthedocs.io/en/stable/containers.html  # docker.models.containers.ContainerCollection.run"  - docker_kwargs:  image: 'head-neck-oars-segmentation:latest' # Docker container tag  network_disabled: True # Disable network  gpu: True # Execute with GPU acceleration  input_mounts: # "src" is reserved keyword for entrypoint for DICOM files  src: /input  Output_mounts: # "dst" is reserved keyword for output DICOM files  dst: /output  pull_before_exec: True # Try pull image before exec. Can be used to # update local image in case a newer container # build exists # DICOM node to receive all DICOM files. destinations:  - host: "X.X.X.X" # May be IP or FCN hostname  port: 104  ae_title: TPS |
| --- |

##

## Supplementary figure S3: Multi modality auto-segmentation

The flow definition for the PROSA trial. The flow is triggered when MR, CT and PET are received simultaneously.

| name: "PROSA-GTV" # Arbitrary name version: "1.3" # Arbitrary version priority: 0 # Priority of this flow. Higher will execute sooner triggers: # List of triggers - Modality: ["MR"] # All dicom files where modality contains "MR" - Modality: ["CT"] # All dicom files where modality contains "CT" - Modality: ["PT"] # All dicom files where modality contains "PT"  # Files will be served to "src" (seeinput_mounts) in a directory tree of # SOPClassUID -> SeriesInstance UID tar_subdir:  - SOPClassUID  - SeriesInstanceUID  # List of all models used in the flow. Only one model here.  models:  - docker_kwargs: # Docker specifications  image: example_prosa_gtv:v1.3 # Docker container tag  gpu: True # This model requires GPU  input_mounts:   src: /input # The DICOM files input  output_mounts:  dst: /output # Every DICOM file in "dst" (/output) will be # sent to # all destinations (see below) pull_before_exec: False # local container - do not pull before exec.  # DICOM node to receive all DICOM files.  destinations:  - host: "X.X.X.X"  port: 104  ae_title: "TPS" |
| --- |

## Supplementary figure S4: Synthetic CT generation

The flow definition for synthetic CTs generation. The flow is triggered when two specified CT types are received along with a registration file.

| name: CBCT to sCT version: "1.0" priority: 0 triggers:  # Planning/Control CT  - Manufacturer: ["Siemens"]  SOPClassUID: ["1.2.840.10008.5.1.4.1.1.2"]  StudyDescription: ["Neck"]  SeriesDescription: ["CycleCUT"]  # CBCT  - Manufacturer: ["Varian"]   SOPClassUID: ["1.2.840.10008.5.1.4.1.1.2"]  # Registration  - SOPClassUID: ["1.2.840.10008.5.1.4.1.1.66.1"]   tar_subdir:  - SOPClassUID  - SeriesDate  models:  - docker_kwargs:  image: local_research_build:latest  gpu: True  input_mounts:  src: /input  output_mounts:  dst: /output_dcm/sCT_CT_FOV # Custom output folder  destinations:  - host: "X.X.X.X"  port: 104  ae_title: TPS |
| --- |

##

## Supplementary figure S5: Asynchronous multi-modality auto-segmentation

An example of how the presented multi modality segmentation flow (S3) may be decomposed as a directed acyclic graph. There are two keywords (“src” and “dst”) of input and output mounts which each may be used exactly one. They denote the entry- and exit node of the graph. All other keys in output mounts must be used as input mount in a different model.

| name: "Async PROSA-GTV" version: "2" priority: 0 triggers:  - Modality: ["MR"]  - Modality: ["CT"]  - Modality: ["PT"]  tar_subdir:  - SOPClassUID  - SeriesInstanceUID  models:  # Converts DICOM files in 'src' to nifti in 'nii' and passes through  # the CT scan to 'dcm' to generate RTSTRUCT later  - docker_kwargs:  image: dicom_to_nifti:example  input_mounts:  src: /input  output_mounts:  nii: /output_nii  ct_dcm: /output_dcm    # Time consuming CPU registration  - docker_kwargs:  image: deformable_registration:example  gpu: False  input_mounts:  nii: /input  output_mounts:  registered_nii: /output    # GPU based target structure prediction with fold 0  - docker_kwargs:  image: prosa_target_segmentation:example  environment:  - FOLD=0  gpu: True  input_mounts:  registered_nii: /input  output_mounts:  segmentation_0: /output  # GPU based target structure prediction with fold 1  - docker_kwargs:  image: prosa_target_segmentation:example  environment:  - FOLD=1  gpu: True  input_mounts:  registered_nii: /input  output_mounts:  segmentation_1: /output   # GPU based target structure prediction with fold 2  - docker_kwargs:  image: prosa_target_segmentation:example  environment:  - FOLD=2  gpu: True  input_mounts:  registered_nii: /input  output_mounts:  segmentation_2: /output   # GPU based target structure prediction with fold 3  - docker_kwargs:  image: prosa_target_segmentation:example  environment:  - FOLD=3  gpu: True  input_mounts:  registered_nii: /input  output_mounts:  segmentation_3: /output   # GPU based target structure prediction with fold 3  - docker_kwargs:  image: prosa_target_segmentation:example  environment:  - FOLD=4  gpu: True  input_mounts:  registered_nii: /input  output_mounts:  segmentation_4: /output  # Ensembles the content of /input*  - docker_kwargs:  image: nifti_segmentation_ensemble:example  gpu: False  input_mounts:  segmentation_0: /input0  segmentation_1: /input1  segmentation_2: /input2  segmentation_3: /input3  segmentation_4: /input4  output_mounts:  ensembled_segmentation: /output   # Converts ensembled segmentation to DICOM RT Structure Set  - docker_kwargs:  image: nifti_to_rtstruct:example  gpu: False  input_mounts:  ensembled_segmentation: /input  output_mounts:  dst: /output  destinations:  - host: "X.X.X.X"  port: 104  ae_title: "TPS" |
| --- |

## A Note on the Medical Device Regulation

In 2017, the European Union (EU) introduced the Medical Device Regulation (MDR), 2017/745 [[2]](https://www.zotero.org/google-docs/?J8QGYk). According to MDR article 2.1, software is an active medical device if it is used for: *“diagnosis, prevention, monitoring, prediction, prognosis, treatment or alleviation of disease”* and in MDCG 2019-11 - Guidance on Qualification and Classification of Software in Regulation (EU) 2017/745 – MDR and Regulation (EU) 2017/746 – IVDR [[3]](https://www.zotero.org/google-docs/?9HNMGI) it is specified under 3.3 decision step 3 that: *“if the software does perform an action on data, or performs an action beyond storage, archival, communication [20], simple search, lossless compression (i.e. using a compression procedure that allows the exact reconstruction of the original data) then it may be a medical device software”.* Further specified in 3.1 then: “*A task such as e-mailing, web or voice messaging, data parsing, word processing, and back-up is by itself not considered as having a medical purpose.”*

According to this, RadDeploy is by the authors considered not to be within the scope of MDR, as the operations do not go beyond storage archival, communication and data parsing. The software put inside model containers might be medical devices and should be classified with annex VIII chapter III of MDR 2017/745 and handled accordingly.

# References

[[1] Home · Wiki · Aarhus RadOnc AI / repos / RadDeploy · GitLab. GitLab 2024. https://gitlab.com/aarhus-radonc-ai/repos/raddeploy/-/wikis/home (accessed May 30, 2024).](https://www.zotero.org/google-docs/?36LLCp)

[[2] Regulation (EU) 2023/607 of the European Parliament and of the Council of 15 March 2023 amending Regulations (EU) 2017/745 and (EU) 2017/746 as regards the transitional provisions for certain medical devices and in vitro diagnostic medical devices (Text with EEA relevance). vol. 080. 2023.](https://www.zotero.org/google-docs/?36LLCp)

[[3] EU MDCG. MDCG 2019-11 Guidance on Qualification and Classification of Software in Regulation (EU) 2017/745 – MDR and Regulation (EU) 2017/746 – IVDR 2019.](https://www.zotero.org/google-docs/?36LLCp)
